# Response

**DOI:** 10.1016/j.chpulm.2024.100086

**Published:** 2024-07-15

**Authors:** Adam J. Brownstein, Christopher B. Cooper, Sonia Jasuja, Alexander E. Sherman, Rajan Saggar, Richard Channick

**Affiliations:** Division of Pulmonary and Critical Care Medicine, University of California, Los Angeles

To the Editor:

We appreciate the comments regarding our research published in *CHEST Pulmonary*.^1^ The author raises important questions that we subsequently address.

Our finding that thermodilution (TD) tended to underestimate cardiac output (CO) compared with direct Fick (DF) in pulmonary hypertension (PH) has the potential to alter clinical management in several clinical scenarios. First, the 2022 PH guidelines^2^ recommend incorporating hemodynamic variables reliant on an accurate CO calculation, including cardiac index and stroke volume index, into risk assessment when initiating therapy. Second, because many centers assess hemodynamic responses to therapy to guide treatment decision-making, an underestimate of cardiac index or stroke volume index may prompt clinicians to unnecessarily escalate PH therapy. Third, an accurate pulmonary vascular resistance is integral in determining eligibility for liver transplantation in portopulmonary hypertension. These scenarios highlight instances in which an underestimation of CO may inappropriately alter clinical management. Alternatively, if DF CO is overestimated due to an inaccurate oxygen consumption ($\dot{V}$o_2_) measurement, hemodynamic risk may be underestimated.

Accurate assessment of $\dot{V}$o_2_ is critical in the determination of DF CO. A metabolic cart measures $\dot{V}$o_2_ in a breath-by-breath analysis. To achieve a steady state, a 2015 systematic review recommends a minimum measurement duration of 4 min (assuming ≤ 10% coefficient of variation in $\dot{V}$o_2_ and carbon dioxide production [$\dot{V}$co_2_]) after discarding the first 5 min of data.^3^ If a steady state is not achieved, an overestimation of $\dot{V}$o_2_ may contribute to a falsely elevated DF CO. It is important to acknowledge that the studies supporting these recommendations did not include patients with PH, which is commonly associated with hyperventilation at rest,^4^^,^^5^ perhaps increasing the chance of not achieving steady state.

The strong concordance in hemodynamic risk assessment between TD and DF indicates that for most patients with PH, TD is adequate. On the contrary, the discrepancy in 20% of patients with PH reinforces the importance of applying a comprehensive approach to risk stratification for accurate assessment of PH severity.

Given that longitudinal studies evaluating the performance of DF compared with TD for predicting clinical outcomes are lacking and that hemodynamic cutoffs proposed in the 2022 guidelines are largely based on TD measurements, relying on TD in clinical practice is appropriate. However, if there is discordance between TD-derived measurements and other variables used for risk assessment, caution should be applied in relying on a single hemodynamic parameter to guide clinical decision-making.

## Financial/Nonfinancial Disclosures

See earlier cited article for author conflicts of interest.
